# Genital self-mutilation in nonpsychotic heterosexual males: Case report of two cases

**DOI:** 10.4103/0019-5545.44753

**Published:** 2008

**Authors:** Rajendra B. Nerli, Indupur R. Ravish, Shrishailesh S. Amarkhed, Ujjaini D. Manoranjan, Vikram Prabha, Ashish Koura

**Affiliations:** Department of Urology, KLES Kidney Foundation, Nehru Nagar, Belgaum - 590 010, India

**Keywords:** Genital self-mutilation, penis, wounds and injuries

## Abstract

Genital self-mutilation is a rare event that is commonly associated with psychotic disorders. However such injuries have also been reported from nonpsychotic patients as a result either from bizarre autoerotic acts, attempts at crude sex change operation by transsexuals or secondary to complex religious beliefs and delusions regarding sexual guilt. We report two cases of genital self-mutilation in nonpsychotic married heterosexual males as a result of conflict and frustration.

## INTRODUCTION

Genital self-mutilation is a severe form of self-injurious behavior. Self-mutilation is described as the “deliberate destruction or alteration of body tissue without conscious suicidal intent.” It has been performed by individuals throughout history. Genital self-mutilation has been a religious practice since ancient Roman times. Roman priests regarded this custom as “a supreme sacrifice of sexual life in favor of the emotion to the highest known good.” Majority of cases of genital self-mutilation reported in the literature have been in patients with psychosis. Greilsheimer and Groves[1] in a group of 52 cases of genital self-mutilation, found 87% to be psychotic and 13% to be nonpsychotic. The psychotic cases ranged from those with functional psychosis to those with brain damage. The nonpsychotic cases included character disorders, transvestism, and complex religious or cultural beliefs. Aboseif *et al*.[2] in a series of 14 patients of self-inflicted genital injuries, found 65% of cases to be psychotic and 35% to be nonpsychotic. It is suggested that genital self-mutilation may be a pathway out of diverse psychological disorders and in nonpsychotic cases it could be an expression of psychotic solution to a conflict and may be influenced by cultural factors.

We report two cases of genital self-mutilation, wherein the patients sought a psychotic solution to a conflict /stress and was influenced by social, cultural, and religious factors.

## CASE REPORT

A 52 year old policeman was admitted with history of genital self-mutilation. This patient was married, had two grown-up children, and had no history of psychosis previously or treatment for the same. The policeman was arrested for some illegal departmental activity and was imprisoned. He was being questioned regarding theft in police armory and was under severe stress. The patient also gave history of severe physical and mental torture. He took this extreme step of self-mutilation as a protest to the stress he was undergoing. He was brought to the hospital in a serious condition. He had mutilated his genitals with a blade. Both the testes and the shaft of penis were severed. The penis was collected from the toilet and brought to the hospital a few hours later. Microvascular reimplantation of the penis was done by the plastic surgeons. The patient was counseled. The penis became ischemic and needed amputation with creation of perineal urethrostomy. The patient was followed up and in this period he needed no psychiatric treatment. The patient died after 34 months due to myocardial infarction.

A 38 year old married construction contractor was admitted with shock due to self-mutilation of genitals. The patient had used a kitchen knife to severe his genitals. As the patient was admitted nearly ten hours after the injury, no attempt for microsurgical reimplantation was attempted. The penile stump was refashioned and the scrotum sutured [Figure 1A and Figure 1B]. The patient was counseled. This patient gave no history of previous psychosis or treatment for the same. This patient was under severe mental stress due to harassment from police and other authorities following the death of a laborer during construction activities. The patient has been under follow-up since 14 months and now desires reconstruction of phallus.

**Figure 1A:**
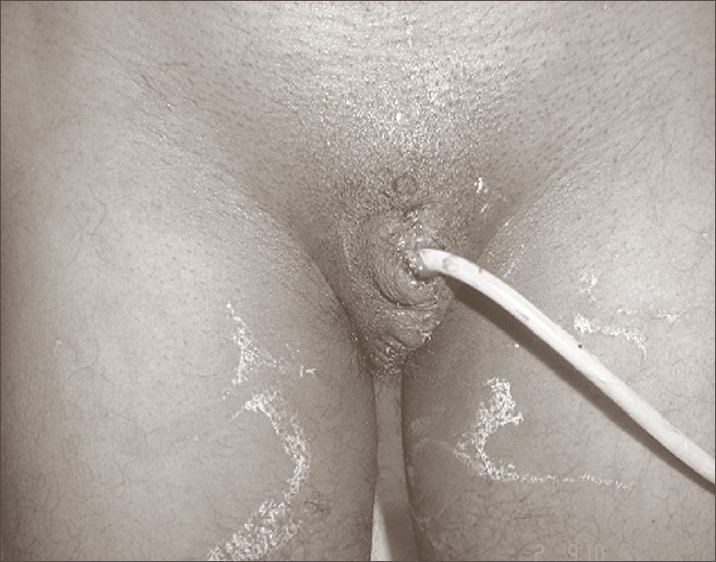
Post-operative photograph following revision of the penile stump

**Figure 1B:**
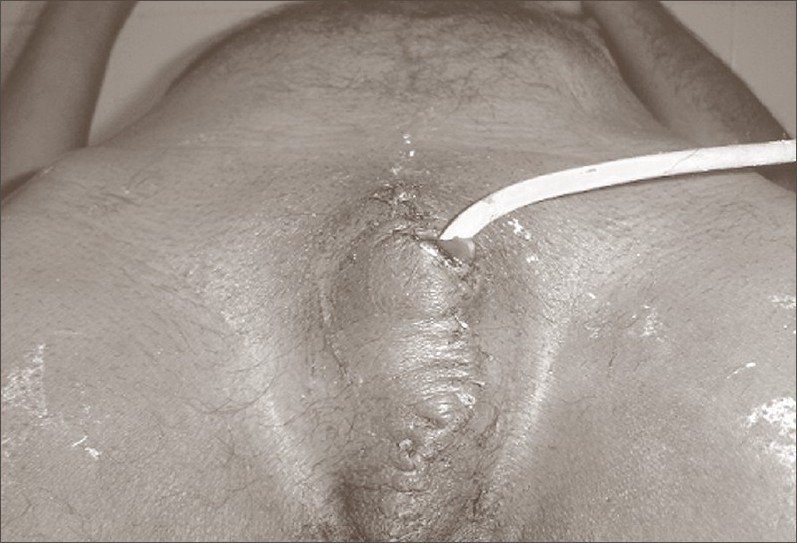
Post-operative photograph following revision of the penile stump and closure of scrotal wound

## DISCUSSION

The most common self-mutilating behavior is cutting one's own wrist which is usually committed by the adolescent or by the mentally retarded, whether for attention seeking purposes or for resolving tension, or as an epidemic behavior. Rarely, self-mutilation has a serious nature, in which the patient attempts to amputate his genital organ, castrate himself, extract his eye or amputate his hand. Such cases are observed in schizophrenia or depression with sexual problems; however, it is sometimes difficult to diagnose the case, because such a behavior is usually the only presenting symptom of the psychiatric disorder.[3] Occasionally, patients attempt self-mutilation under the influence of an ordering hallucination.[3] This phenomenon is called the “van Gogh syndrome,” named after Vincent van Gogh, the impressionist painter who cut his ear to dedicate it to his beloved one.[3]

The majority of cases of genital self-mutilation reported in the literature have been in patients with psychosis or psychiatric disorders,[4] with either functional or organic brain disease.[1,5] However, a few cases have been described in nonpsychotic persons and result either from bizarre autoerotic acts[6] or from attempts at crude sex change operation by the transsexuals.[7] A verse suggesting autocastration is found in Mathew 19:11 “For these are some eunuchs, which were made eunuchs of men, and there be eunuchs which have made themselves eunuchs for the kingdom of heaven's sake.” Ames[8] suggested the eponym of the Klingsor syndrome to apply to the occurrence of autocastration as a consequence of religious delusions.[9] Thus, self-mutilation of the male genitals seems to be a pathway out of diverse psychological disorder or behavior and cultural beliefs.

It is not clear what prompts a man to divest himself of his own genitalia. Greilsheimer and Groves[1] were able to identify two general groupings in their series of 52 cases of genital self-mutilation. The most common group consisted of psychotic patients (87%). The other group consisted of nonpsychotic persons with character disorders, transvestites who anticipated their own gender conversion surgery or patients with complex religious or cultural beliefs. Aboseif *et al*.[2] in their series reported 65% of their patients belonging to the psychotic group and the remaining 35% to the nonpsychotic group. Of the patients with no clear psychotic illness: three were transsexuals, one was homosexual and one was heterosexual. There was no difference in the severity of the injury between the two groups. A history of substance abuse (excessive alcohol intake and/or narcotics) was present in 54% of their patients.

Sudarshan *et al*.[4] reported a case of self-mutilation in a 29 year old nonpsychotic patient, who chiseled off his penis, because of his sexual inadequacy and if married was scared of the consequences of marriage in such a situation. He was under pressure from his parents to get married and sought a solution to his problem by amputating his penis so as to not get married.

In our cases, both the patients were heterosexual with no history of previous psychosis. Both the patients were assessed and counseled. Their act of self-mutilation of their genitalia could primarily be secondary to conflict, expression of internalized frustration which resulted in an impulsive act of self-injurious attempt.

Management of genital self-mutilation injury has been a challenging problem. Evaluation and treatment of these patients require close collaboration among surgeons, psychiatrists, and experienced medical personnel. Immediate full genital reconstruction should be done regardless of mental status. In addition to the immediate surgical management of the injury, optimal management usually requires long term therapy that aims at correction of the psychiatric illness or containment of ungovernable effects associated with the impulse disorder.
